# Plasminogen Activator Inhibitor-1 Level Correlates with Lipoprotein Subfractions in Obese Nondiabetic Subjects

**DOI:** 10.1155/2018/9596054

**Published:** 2018-05-30

**Authors:** Sándor Somodi, Ildikó Seres, Hajnalka Lőrincz, Mariann Harangi, Péter Fülöp, György Paragh

**Affiliations:** ^1^Department of Internal Medicine, Faculty of Medicine, University of Debrecen, Debrecen, Hungary; ^2^Department of Clinical Pharmacology, Faculty of Pharmacy, University of Debrecen, Debrecen, Hungary

## Abstract

**Background:**

The elevated level of plasminogen activator inhibitor-1 (PAI-1) in obese subjects with metabolic syndrome and in patients with type 2 diabetes is well established. The association of plasma PAI-1 and lipid metabolism is still unclear. The aim of the present study was to determine the relationship between plasma PAI-1 levels and the distribution of lipoprotein subfractions in obese and lean nondiabetic individuals.

**Subjects and Methods:**

We enrolled fifty nondiabetic obese patients and thirty-two healthy volunteers. Lipoprotein subfractions were detected with Lipoprint System. Plasma PAI-1, tumor necrosis factor-*α* (TNF-*α*), interleukin-6 (IL-6), and myeloperoxidase (MPO) concentrations were determined with enzyme-linked immunosorbent assay (ELISA), while serum paraoxonase-1 (PON1) activities were measured by spectrophotometry.

**Results:**

The TNF-*α*, IL-6, oxidized low-density lipoprotein (oxLDL), and MPO levels were found to be significantly higher, while PON1 paraoxonase and arylesterase activities were nonsignificantly lower in the obese patients. Strong significant negative correlations were found between plasma PAI-1 concentration and mean LDL size, as well as between PAI-1 concentrations and the levels of the large and intermediate high-density lipoprotein (HDL) subfractions. In multiple regression analysis, PAI-1 was predicted by waist circumference and intermediate HDL subfraction.

**Conclusion:**

The significant correlations between PAI-1 levels and lipoprotein subfractions indicate the link between PAI-1 and lipid metabolism in obesity.

## 1. Introduction

Excess adipose tissue in obesity is directly associated with increased cardiovascular mortality and, in part, due to comorbidities including hypertension, diabetes, and dyslipidemia [1, 2]. Low-grade inflammation and the procoagulant milieu of obesity also contribute to increased cardiovascular risk [3]. Plasminogen activator inhibitor-1 (PAI-1) is the primary regulator of fibrinolysis breaking down fibrin in the intravascular compartment as well as breaking down the extracellular matrix. PAI-1 also controls cell adhesion and migration in the extravascular space and therefore may influence the development of atherosclerotic plaque formation [4–6]. Elevated PAI-1 concentration is an established risk factor for coronary artery disease [7, 8].

Increased levels of PAI-1 in obese subjects with metabolic syndrome and in type 2 diabetic patients are well documented [9–11]. Furthermore, a direct association was observed between PAI-1 level and waist circumference in healthy subjects [12]. In another study, values of BMI and PAI-1 correlated significantly in obese and overweight subjects [13]. The association between PAI-1 and obesity is considered to be the result of increased PAI-1 production of adipose tissue by various cell types including preadipocytes, mature adipocytes, macrophages, endothelial and smooth muscle cells [14, 15]. It is generally accepted that visceral or ectopic adipose tissue produces more PAI-1 than does subcutaneous adipose tissue [16]. Visceral adipose tissue secretes higher amount of proinflammatory cytokines, such as TNF-*α*, TGF-*β*1, IL-6, and IL-1, and also contains more stromal cells and macrophages, which are the main cellular components of adipose tissue that produce PAI-1 [17, 18].

Plasma lipoproteins represent heterogeneous populations with respect to their size, density, electrophoretic mobility, and lipid and protein composition [19]. Low-density lipoprotein (LDL) particles transport lipid molecules into peripheral tissues, for example, into the arterial wall. Predominance of the small-dense LDL subspecies results in increased susceptibility to oxidative modifications and subsequently enhanced atherosclerosis and increased cardiovascular risk [20]. In turn, high-density lipoprotein (HDL) particles are generally considered to be protective against atherosclerosis, as the large-sized HDL subfractions are primarily ascribed to reverse cholesterol transport [21], while the small-sized HDL subfractions rather exert antioxidant effects through their associated anti-inflammatory enzyme termed paraoxonase-1 (PON1). Myeloperoxidase (MPO) is a leukocyte-derived heme protein bound to HDL and is linked to enhanced oxidative stress and atherosclerosis. Recent data indicate that MPO, PON1, and HDL may bind and inhibit each other, forming a ternary complex [22].

In epidemiological studies, direct associations were observed between PAI-1 and lipid parameters (e.g., triglyceride and HDL-C levels) in healthy young adults [12]. Also, recent studies postulated that very low density lipoprotein (VLDL) was capable of increasing the PAI-1 level through a VLDL response element localized to the promoter region of the PAI-1 gene, mediating VLDL-induced PAI-1 transcription in endothelial cells [23, 24]. A previous *in vitro* study also showed that small-sized HDL, but not large-sized HDL, stimulated PAI-1 release in the murine 3T3 adipocyte cell line [25]; however, to date, there are limited data on the association of PAI-1 with the functional and structural properties of the different lipoprotein fractions. Therefore, the aim of the present study was to examine the relationship between plasma PAI-1 levels and the distributions of HDL and LDL subfractions as well as to confirm the potential associations between the concentrations of PAI-1 and the HDL-linked enzymes PON1 and MPO, respectively, in obese and lean nondiabetic patients.

## 2. Patients and Methods

### 2.1. Study Population

We enrolled fifty nondiabetic obese patients that were referred to our obesity outpatient clinic at the Division of Metabolic Diseases, Department of Medicine, University of Debrecen, Hungary, and thirty-two sex- and age-matched healthy volunteers. All participants provided written informed consent. The study protocol was approved by the Local Ethical Committee of University of Debrecen, and the study was carried out in accordance with the Declaration of Helsinki. Obesity was defined as BMI ≥ 30 kg/m^2^, while the BMI of the control subjects was normal (between 18.5 and 29.9 kg/m^2^). Participants with active liver, endocrine (including any types of diabetes mellitus), cardiovascular, pulmonary, neurological, gastrointestinal, psychiatric, acute infective, or autoimmune disease; renal impairment; or malignancy were excluded. Further exclusion criteria were pregnancy, lactation, current smoking, and alcoholism or drug dependence. Neither obese subjects nor nonobese healthy controls were taking lipid-lowering, hyperglycemic, anti-inflammatory, and antithrombotic medications or dietary supplements and any drug, which may interfere with the measurements. None of the participants were on antihypertensive treatment with the exception of ten obese patients, who were on diuretics (indapamide) because of mild hypertension.

### 2.2. Sample Collection and Laboratory Measurements

All venous blood samples were collected after a 12-hour fasting. The routine laboratory parameters were measured by the Central Laboratory of the University of Debrecen. The measured parameters were the following: fasting glucose, fructosamine, high-sensitivity C-reactive protein (hsCRP), total cholesterol, triglyceride, HDL cholesterol (HDL-C), LDL cholesterol (LDL-C), apolipoprotein AI (apoAI), apolipoprotein B (apoB), and lipoprotein(a). To check the carbohydrate metabolism of the study participants, we applied a routine 75 g oral glucose tolerance test (OGTT) after an overnight fast. At the same time, hemoglobin A1c (HbA1c) and fasting insulin were also measured. Homeostasis model assessment-insulin resistance (HOMA-IR) was calculated with the formula of Matthews et al. [25]. Serum and EDTA plasma samples were kept frozen at −70°C for enzyme-linked immunosorbent assay (ELISA) measurements and for subsequent lipoprotein subfraction analysis. In our work, we followed the methods of Szentpéteri et al. [26].

### 2.3. Lipoprotein Subfraction Analyses

HDL subfractions were analyzed by a polyacrylamide gel-electrophoresis with the Lipoprint System (Quantimetrix Corp., CA, USA) according to the manufacturer's instructions as previously published [27].

Briefly, 25 *μ*l serum of samples was added to the polyacrylamide gel tubes along with 300 *μ*l lipophilic Sudan Black containing a loading gel solution and was photopolymerized at room temperature (20–22°C) for 30 min. Tubes were performed by electrophoresis at a constant of 3 mA/tube for 50 min. Each electrophoresis chamber involved a standard-quality control provided by the manufacturer (Liposure Serum Lipoprotein Control, Quantimetrix Corp., CA, USA). After electrophoresis, stained subfractions were scanned with an ArtixScan M1 digital scanner (Microtek International Inc., CA, USA) and were identified by their mobility (Rf) using VLDL + LDL as the starting (Rf 0.0) reference point and albumin as the ending (Rf 1.0) reference point.

Between the starting and ending reference points, 10 HDL subfraction bands were determined and were collected into three major classes: large (HDL1–HDL3), intermediate (HDL4–HDL7), and small (HDL8–HDL10) HDL subfractions. Cholesterol concentrations of the HDL particles were calculated with Lipoware software (Quantimetrix Corp., CA, USA) by multiplying the total HDL-C concentration of the samples by the relative area under the curve (AUC%) of the subfraction bands.

LDL subfractions were also detected using Lipoprint System (Quantimetrix Corp., CA, USA) [26] according to the instructions of the manufacturer. 25 *μ*l sera were added to polyacrylamide gel tubes along with 200 *μ*l Sudan Black containing a loading gel solution. The sample loading gel mixture was photopolymerized for 30 minutes at 20–22°C prior to electrophoresis at a constant of 3 mA/tube for 60 min.

LDL subfractions were identified after electrophoresis by their mobility (Rf) using VLDL as the reference point (Rf 0.0) and HDL as the ending reference point (Rf 1.0). AUC% for the VLDL, midbands A, B, and C (comprising primarily IDL), up to seven LDL subfractions and HDL peaks were calculated by Lipoware computer software (Quantimetrix Corp., CA, USA). Percentage of large LDL (large LDL%) was defined as the sum of the percentage of LDL1 and LDL2, whereas percentage of small LDL (small-dense LDL%) was defined as the sum of LDL3–LDL7. Cholesterol concentrations of LDL subfractions were determined by multiplying the relative AUC of subfractions by the total cholesterol concentration of the sample. Calculated total LDL-C is comprised of the sum of the cholesterol in midbands (A, B, and C) and LDL subfractions (LDL1–LDL7) and strongly correlates with the directly measured LDL-C [28, 29]. Mean LDL size was calculated by Lipoware software.

### 2.4. Determination of Human Paraoxonase-1 Enzyme Activities

Serum PON1 arylesterase activity was measured with phenylacetate substrate (Sigma Aldrich, Hungary), and the hydrolysis of phenylacetate was monitored at 270 nm at room temperature as previously described [30, 31]. Serum PON1 paraoxonase activity was assayed on a microtiter plate by a kinetic, semiautomated method using paraoxon (O,O-diethyl-O-p-nitrophenyl phosphate, Sigma-Aldrich, Hungary) as a substrate. Hydrolysis of paraoxon was followed at 405 nm at room temperature.

### 2.5. ELISA Measurements

Human plasma serpin E1/PAI-1, TNF-*α*, IL-6, and MPO concentrations were measured by a commercially available ELISA kit (R&D Systems, Minneapolis, MN, USA) according to the manufacturer's instructions. The intra- and interassay coefficients of variations were 3.1–8.7% and 7.2–10.4% (TNF-*α*), 6.9–7.8% and 6.5–9.6% (IL-6), and 6.6–7.7% and 6.5–9.4% (MPO), respectively. Oxidized LDL levels were detected by ELISA (Mercodia AB, Sweden) with 5.5–7.3 CV% intra-assay precision and 4–6.2 CV% inter-assay precision, respectively, according to the recommendations of the manufacturers.

### 2.6. Statistical Methods

Statistical analysis was performed by STATISTICA software, version 8.0 (StatSoft Inc., Tulsa, OK, USA). Data were presented by descriptive analysis (means ± SD in the case of normal distribution, or medians [lower quartile–upper quartile] in the case of nonnormal distribution). The Kolmogorov-Smirnov test was used for testing the normality of data distribution. Comparisons between groups were performed by Student's unpaired *t*-test in the case of normally distributed variables and by the Mann–Whitney *U* test in the case of variables with nonnormal distribution. Correlations between continuous variables were assessed by linear regression analysis using Pearson's test. Since the distribution of some variables of interest became normal upon base-10 logarithm transformation, we used in the case of these variables the log values for correlation analyses. Multiple regression analysis was performed to determine the variables' best-predicted PAI-1 concentrations. Results were considered to be significant at the level of *p* < 0.05.

## 3. Results

Anthropometric and laboratory characteristics of study participants are summarized in Table 1. Obese patients had extremely high BMI (*p* < 0.001) and slightly elevated hsCRP level (*p* < 0.001), compared to lean individuals. Although there were several other differences in the laboratory parameters in nondiabetic obese patients compared to lean controls, these data were found to be in the physiological range. Plasma triglyceride and lipoprotein(a) concentrations were measured to be significantly higher, while the levels of HDL-C and apoAI were significantly lower in the obese group, compared to normal-weight controls. Regarding the carbohydrate parameters, the HbA1c level was significantly higher, but within the normal range in the obese individuals. Fasting glucose was in the normal range in both groups, and blood glucose level at 120 min of OGTT was not elevated in the obese group. The obese patients involved into this study had neither diabetes nor abnormal glucose tolerance on the basis of glucose concentrations during OGTT.

Levels of the inflammatory markers TNF-*α* and IL-6 were significantly higher (*p* < 0.01 and *p* < 0.001, resp.) in nondiabetic obese patients compared to lean individuals (Table 1). Additionally, the levels of oxidized LDL and MPO were found to be significantly higher (*p* < 0.01 and *p* < 0.05, resp.), while PON1 paraoxonase and arylesterase activities were nonsignificantly lower in the obese patients.

Absolute amounts of lipoprotein subfractions are shown in Table 2. The concentration of the VLDL subfraction was measured to be higher (*p* < 0.001), while IDL levels were lower in the obese patients compared to controls (*p* < 0.001). Levels of large and small-dense LDL subfractions were increased in the patient group, but not in the lean participants (*p* < 0.01 and *p* < 0.001, resp.). Mean LDL size was significantly lower in the obese subjects (*p* < 0.001). Although HDL-C levels fell into the normal range in both studied groups, there was a shift towards the small-sized HDL particles in nondiabetic obese individuals. The concentrations of the large and intermediate HDL subfractions were found to be significantly decreased (*p* < 0.001 and *p* < 0.05, resp.), while small HDL subfraction levels were significantly increased in the obese patients compared to the normal-weight individuals (*p* < 0.01) (Table 2).

**Figure 1:**
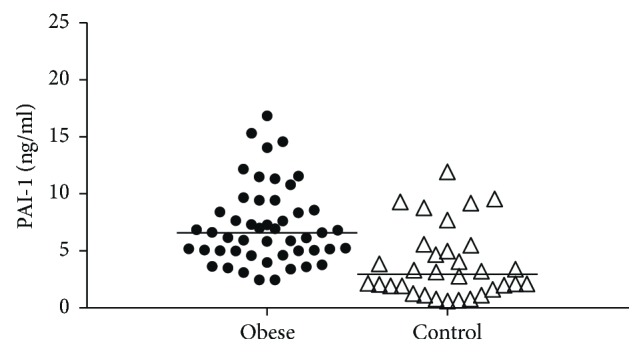
Plasma concentrations of plasminogen activator inhibitor-1 (PAI-1) in nondiabetic obese and lean participants (obese: 6.58 [5–8.58] and control: 2.93 [1.8–5.23] ng/ml; *p* < 0.001).

**Figure 2:**
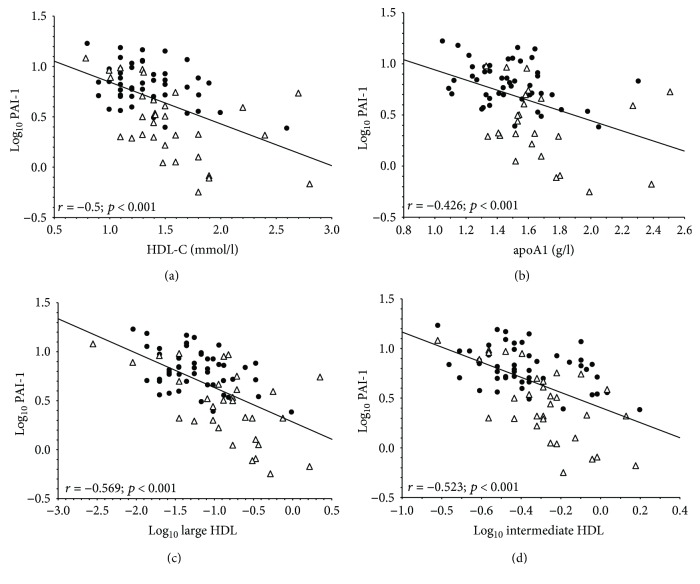
Correlations between plasminogen activator inhibitor-1 (PAI-1) and (a) high-density lipoprotein cholesterol (HDL-C), (b) apolipoprotein AI (apoAI), (c) large HDL subfraction, and (d) intermediate HDL subfraction in nondiabetic obese (●) and normal-weight controls (∆).

The median level of PAI-1 was also significantly higher in obese patients compared to lean participants (obese: 6.58 [5–8.58] versus control: 2.93 [1.8–5.23] ng/ml; *p* < 0.001) (Figure 1). We were unable to detect gender differences between females and males neither in the obese (*p* = 0.458) nor in the control group (*p* = 0.314), respectively. Pearson correlations of PAI-1 concentrations with the lipid parameters of the study participants are summarized in Table 3. BMI, waist circumference, triglyceride, VLDL subfraction, large LDL subfraction, and small-dense LDL subfraction showed positive associations with PAI-1 levels, while there was an inverse correlation between the concentrations of the IDL subfraction and PAI-1, as well as between mean LDL size and PAI-1 level. Plasma PAI-1 concentrations correlated negatively with total HDL-C and apoA1 levels, respectively, in the whole study population (Table 3; Figures 2(a) and 2(b)). We also found a strong inverse correlation between plasma PAI-1 levels and the concentrations of large and intermediate HDL subfractions, respectively (Table 3; Figures 2(c) and 2(d)).

Furthermore, plasma PAI-1 levels showed positive correlations with hsCRP, IL-6, and TNF-*α* levels in the study participants, whereas oxidized LDL and MPO concentrations, as well as PON1 paraoxonase and arylesterase activities, did not show associations with plasma PAI-1 levels (Table 3).

To test whether the associations detected in univariate analyses were independent of lipid parameters, we carried out a multiple regression analysis with PAI-1 level as the dependent variable. Besides age, gender, BMI, and waist circumference, the model included large HDL, intermediate HDL, small HDL, large LDL, and small-dense LDL subfraction concentrations and mean LDL size. Based on the backward stepwise analysis, the PAI-1 level turned out to be best predicted by waist circumference and intermediate HDL subfraction concentration (Table 4).

## 4. Discussion

To the best of our knowledge, this is the first report about the correlations between plasma PAI-1 levels and lipoprotein subfractions *in vivo*, indicating the potential role of disturbed lipid metabolism in PAI-1 overproduction in obesity. Levels of other inflammatory markers (CRP, IL-6, TNF-*α*, oxLDL, and MPO) were increased in the obese nondiabetic group as well, while HDL-bound antioxidant enzyme activities (PON1 paraoxonase and arylesterase) were nonsignificantly lower, compared to the lean controls.

In line with a previous study [32], higher levels of VLDL subfractions were found in the obese patients while the LDL-C levels did not differ significantly between the obese and control groups. Lipoprotein subfraction analysis showed a decreased mean LDL size and higher amounts of large and small-dense LDL subfractions contributing to the predominance of the atherogenic lipid profile in obesity [20]. While epidemiological studies reported decreased HDL-C concentrations in obese patients, the atherogenic properties of the various HDL subfractions are contradictory because of their functional and structural heterogeneity [33]. Previous studies also indicated that decreased amount of large HDL particles and increased amount of small HDL particles were closely and significantly associated with increased cardiovascular risk [21]. However, a prospective study performed on type 2 diabetes patients reported that the level of large-sized, but not the small-sized HDL subfraction predicted a lower risk of cardiovascular disease [34]. In our obese nondiabetic group, the HDL-C concentration was in normal range but it was significantly lower compared to the lean controls. Additionally, HDL subfraction analysis showed higher concentrations of small HDL and lower levels of intermediate- and large-HDL subfractions in the obese nondiabetic group compared to the lean controls. The observed differences in the distribution of the lipoprotein subfractions may highlight the importance of the early screening of obese individuals to predict the development of subsequent cardiovascular complications.

The link between increased circulating PAI-1 level and metabolic syndrome is not completely clarified. PAI-1 concentration was found to be strongly associated with the components of metabolic syndrome (degree of obesity, elevated triglyceride level, decreased HDL level, and insulin resistance) [35]; however, other studies did not find an association between plasma PAI-1 level and dyslipidemia [36, 37]. These findings suggest that plasma PAI-1 concentration is rather determined by the phenotypic distribution of the adipocytes, but it is not closely dependent on total fat mass. Indeed, plasma PAI-1 is mostly considered as a marker of ectopic fat deposition [16] and although both BMI and waist circumference showed significant pairwise correlations with PAI-1 level in our study, only the waist circumference remained the predictor of PAI-1 level among anthropometric parameters during the multivariate analysis.

There are many mediators that may play a significant role in the interaction between PAI-1 and the traits of metabolic syndrome. Correlations between TNF-*α* and TGF-*β* pathways and PAI-1 in adipose tissue are well documented [38, 39]. Dexamethasone and cortisol are potent inducers of PAI-1 synthesis in cultured adipocytes; therefore, cortisol may also be an inducer of PAI-1 in visceral obesity [40]. Hypertriglyceridemia and VLDL also induce PAI-1 synthesis through a VLDL response element localized in the promoter region of the PAI-1 gene [23]. Since we did not investigate these associations, we cannot state whether such mechanisms exist in nondiabetic obese individuals *in vivo.*

We could find gender differences in PAI-1 level neither of obese nor of lean subjects. The gender difference of plasma PAI-1 level is controversial. Margaglione and coworkers found elevated PAI-1 level in males in a general population. The male sex was an independent variable determining PAI-1 level in multiple stepwise regression analysis [41]. van Harmelen et al. have found two times higher plasma PAI-1 activity in males, which was mainly due to gender differences in the waist-hip ratio. [42]. Mantovani et al. reported gender differences in PAI-1 levels of adolescents. In normal-weight males, PAI-1 levels were significantly lower than those of the females [43]. Based on our backward stepwise analysis, the PAI-1 level was not predicted by gender (Table 4).

Corroborating previous findings, a significant positive correlation was detected between PAI-1 and triglyceride levels, while PAI-1 and HDL-C showed a negative correlation in nondiabetic obese patients and lean controls. Additionally, we demonstrated a strong, significant negative correlation between mean LDL size and plasma PAI-1 concentration. Furthermore, we detected a significant positive correlation between the most atherogenic, small-dense LDL subfraction concentration and plasma PAI-1 level. The negative correlation between plasma PAI-1 level and LDL size was described by Festa et al. in a nondiabetic population, while this association was weaker both in patients with impaired glucose tolerance and in diabetic ones [44]. Additionally, a positive association was found between the levels of plasma PAI-1 and small-dense LDL in healthy middle-aged men [45]. This is the first work, however, to evaluate the correlations between LDL size/small-dense LDL levels and plasma PAI-1 concentrations in obese nondiabetic patients. These data support the suggested link between LDL and PAI-1 in accelerated atherogenesis and increased cardiovascular risk of obese patients; however, our hypothesis needs to be verified in further investigations.

The levels of large- and intermediate-HDL subfraction showed negative associations with the circulating PAI-1 levels, but no correlation was found between the PAI-1 level and the concentration of small HDL subfraction in the whole cohort. Based on the multivariate analysis, waist circumference and intermediate HDL subfraction are the independent predictors of plasma PAI-1 level; however, the link between HDL subfractions and plasma PAI-1 level is not well understood. Using sequential ultracentrifugation, Lee et al. showed that HDL3-, but not HDL2-bound sphingosine-1-phosphate, stimulates PAI-1 secretion in a concentration-dependent manner in 3T3 adipocytes. In our study, we could not find a significant correlation between PAI-1 and small-HDL subfraction concentration (which mostly corresponds to HDL3) using nongradient gel electrophoresis for lipoprotein subfraction analysis. Still, based on the *in vitro* results, sphingosine-1-phosphate may have a potential role in elevated plasma PAI-1 level in obese nondiabetic patients [46]. Further studies need to clarify the link between PAI-1 production and sphingosine-1-phosphate in obese patients.

We are aware of some limitations of this study. The relatively small size of the study population and the small number of the enrolled males may reduce the power of our study; however, the significant correlations between PAI-1 levels and lipoprotein subfractions underline the association between PAI-1 and lipid metabolism. Beside quantitative measurement of lipoprotein subfractions using Lipoprint, determination of qualitative properties of lipoproteins, that is, cholesterol efflux capacity, may provide more detailed data about impaired lipoprotein function in obesity. Moreover, a more precise assessment of visceral and ectopic fat mass (MRI or PET scan) should be also helpful for the evaluation of the association between PAI-1 level and the amount of fat storage.

## 5. Conclusion

In conclusion, we found a strong significant negative correlation in obese and lean subjects between plasma PAI-1 concentration and the levels of large- and intermediate-HDL subfractions. Mean LDL size showed a negative correlation with plasma PAI-1 concentration. On the basis of multiple regression analysis, waist circumference and intermediate-HDL subfraction were predictors of plasma PAI-1 concentration. In contrast with earlier reports, the above correlations underline the relationship between PAI-1 and lipoprotein metabolism in obesity. Further studies on obese patients with diabetes or coronary heart disease might also add valuable information about the relationship of PAI-1 and obesity-related lipid abnormalities.

## Figures and Tables

**Table 1:** Anthropometric and routine laboratory parameters of the study participants.

	Obese (*n* = 50)	Control (*n* = 32)	*p*
Gender (F/M)	43/7	27/5	ns
Age (yr)	44.20 ± 13.50	41.78 ± 5.97	ns
Body mass index (kg/m^2^)	41.96 ± 8.63	24.47 ± 2.51	<0.001
Waist circumference (cm)	119.76 ± 16.87	83.62 ± 9.25	<0.001
hsCRP (mg/l)	8.24 (3.2–13.09)	1.57 (0.6–2.94)	<0.001
Fructosamine (*μ*mol/l)	225.32 ± 27.95	229.0 ± 11.65	ns
Thyroid stimulating hormone (mU/l)	1.98 ± 0.98	1.93 ± 1.15	ns
Lipid parameters			
Triglyceride (mmol/l)	1.4 (1.1–2.0)	1.0 (0.75–1.39)	<0.01
Total cholesterol (mmol/l)	5.04 ± 0.83	5.07 ± 0.78	ns
HDL cholesterol (mmol/l)	1.36 ± 0.33	1.56 ± 0.46	<0.05
LDL cholesterol (mmol/l)	3.17 ± 0.74	2.93 ± 0.52	ns
Apolipoprotein A–I (g/l)	1.48 ± 0.24	1.68 ± 0.31	<0.01
Apolipoprotein B (g/l)	0.86 ± 0.20	0.94 ± 0.18	ns
Lipoprotein(a) (mg/l)	248 (120–586)	70 (30–214)	<0.001
Carbohydrate parameters			
Hemoglobin A1C (%)	5.76 ± 0.54	5.07 ± 0.33	<0.001
Fasting glucose (mmol/l)	4.90 ± 0.75	4.82 ± 0.48	ns
OGTT 120 min	7.00 ± 2.01		
Fasting insulin (mU/l)	21.01 ± 15.91		
HOMA-IR	3.75 (2.4–6.52)		
Inflammatory and oxidative markers			
Tumor necrosis factor-*α* (pg/ml)	1.783 (1.41–2.254)	1.39 (1.20–1.67)	<0.01
Interleukin-6 (pg/ml)	3.439 (1.817–4.89)	0.97 (0.63–1.28)	<0.001
Oxidized LDL (U/l)	46.8 ± 9.95	41.1 ± 9.57	<0.01
Paraoxonase activity (U/l)	64.72 (43.79–149.52)	83.03 (47.9–167.4)	ns
Arylesterase activity (U/l)	121.61 ± 23.65	131.1 ± 28.75	ns
Myeloperoxidase (ng/ml)	280 (148.3–386.3)	207.9 (125.8–265.2)	<0.05

Values are presented as *mean* ± *standard* deviation or median (lower quartile–upper quartile). HDL: high-density lipoprotein; hsCRP: high-sensitivity C-reactive protein; LDL: low-density-lipoprotein; OGTT: oral glucose tolerance test; HOMA-IR: homeostasis model assessment-insulin resistance; ns: nonsignificant.

**Table 2:** Concentrations of lipoprotein subfractions in nondiabetic obese and lean participants.

	Obese (*n* = 50)	Control (*n* = 32)	*p*
VLDL subfraction (mmol/l)	1.165 ± 0.17	0.868 ± 0.17	<0.001
Midband (IDL) (mmol/l)	1.207 ± 0.31	1.505 ± 0.38	<0.001
LDL subfractions			
Large LDL (mmol/l)	1.267 (1.06–1.603)	1.047 (0.827–1.344)	<0.01
Small-dense LDL (mmol/l)	0.091 (0.026–0.155)	0.026 (0–0.052)	<0.001
Mean LDL size (nm)	26.98 ± 0.31	27.26 ± 0.37	<0.001
HDL subfractions			
Large HDL (mmol/l)	0.284 (0.207–0.388)	0.453 (0.31–0.608)	<0.001
Intermediate HDL (mmol/l)	0.6594 (0.595–0.828)	0.749 (0.659–0.853)	<0.05
Small HDL (mmol/l)	0.336 (0.284–0.388)	0.284 (0.246–0.336)	<0.01

Values are presented as *mean* ± *standard* deviation or median (lower quartile–upper quartile).

**Table 3:** Linear regression analyses of log_10_ PAI-1 in all study participants.

Variable	*r*	*p*
Age	0.098	0.38
Body mass index	0.578	<0.001
Waist circumference	0.579	<0.001
Lipid parameters		
Log_10_ triglyceride	0.421	<0.001
Total cholesterol	−0.167	0.14
VLDL subfraction	0.284	0.01
Midband (IDL) subfraction	−0.482	<0.001
LDL-C	0.074	0.51
Apolipoprotein B	−0.078	0.51
Log_10_ large LDL subfraction	0.382	<0.001
Log_10_ small-dense LDL subfraction	0.315	0.01
Mean LDL size	−0.447	<0.001
HDL-C	−0.5	<0.001
Apolipoprotein AI	−0.426	<0.001
Log_10_ large HDL subfraction	−0.569	<0.001
Log_10_ intermediate HDL subfraction	−0.523	<0.001
Log_10_ small HDL subfraction	0.177	0.11
Inflammatory markers		
Log_10_ hsCRP	0.444	<0.001
Log_10_ interleukin-6	0.485	<0.001
Log_10_ tumor necrosis factor-*α*	0.223	<0.05
Oxidative stress markers		
Oxidized LDL	0.171	0.13
Log_10_ PON1 paraoxonase activity	−0.157	0.16
PON1 arylesterase activity	−0.194	0.09
Log_10_ myeloperoxidase	0.129	0.26

HDL: high-density lipoprotein; hsCRP: high-sensitivity C-reactive protein; LDL: low-density lipoprotein; PON1: paraoxonase-1.

**Table 4:** Multivariate analysis in all study participants for PAI-1 as a dependent variable.

Variable	Beta	*p* value
Age	0.025	ns
Gender	0.254	ns
BMI	−0.26	ns
Waist circumference	0.342	<0.001
Log_10_ large HDL	−0.02	ns
Log_10_ intermediate HDL	−0.52	<0.001
Log_10_ small HDL	0.093	ns
Log_10_ large LDL	−0.03	ns
Log_10_ small-dense LDL	−0.09	ns
Mean LDL size	−0.15	ns
